# Donor-derived airway air–liquid interface model for high-throughput screening of antiviral combinations with concurrent analysis of antiviral efficacy and epithelial toxicity using ciliR

**DOI:** 10.1183/23120541.01283-2025

**Published:** 2026-06-01

**Authors:** Daniela Cardinale, Oriane Grant, Dani Do Hyang Lee, Samuel Ellis, Robert E. Hynds, Richard Angell, Chris O'Callaghan, Mario Cortina-Borja, Elaine Thomas, Claire M. Smith

**Affiliations:** 1 UCL Great Ormond Street Institute of Child Health, London, UK; 2 UCL Cancer Institute, University College London, London, UK; 3 Translational Research Office, UCL School of Pharmacy, London, UK; 4 Anti-infectives Research Unit, Pfizer, UK; 5 These authors contributed equally

## Abstract

**Background:**

Respiratory RNA viral infections significantly impact quality of life and productivity, in terms of both pandemics (*e.g.* severe acute respiratory syndrome coronavirus 2, influenza A viruses) and seasonal infections, such as respiratory syncytial virus (RSV). Globally, there are an estimated 33.8 million cases of RSV annually in children aged <5 years, leading to 2.8–4.3 million hospital admissions and up to 199 000 deaths. Human-relevant drug screening models are key to addressing the urgent need for effective antiviral treatments for RSV, particularly in vulnerable patient groups.

**Methods:**

Here, we present a donor-derived differentiated primary human nasal epithelial cell, high-throughput screening (HTS) Transwell-96 air–liquid interface (ALI) model designed to investigate the effects of combination antiviral therapies on RSV infection in primary human ciliated airway epithelium. In addition, we describe novel analytical tools using R (ciliR) to screen drug combinations by concurrently measuring efficacy and ciliary beat frequency, which we used as a sensitive marker of cell toxicity.

**Results:**

Our results demonstrate that the higher-throughput HTS Transwell-96 ALI cultures retain epithelial composition and ciliary function consistent within donor replicates. These cultures are permissible to infection with an RSV-green fluorescent protein (GFP) reporter virus, enabling quantitative comparison and combination treatment across multiple epithelial cultures from the same donor.

**Conclusion:**

We anticipate that our disease-relevant system will serve as a foundation for larger-scale experiments aimed at optimising combination therapy for RSV and other respiratory viruses.

## Shareable abstract

**96-well differentiated epithelial cell model and R-based analysis pipeline (ciliR, SynergyFinder) enable parallel quantification of RSV antiviral efficacy and epithelial toxicity – paving the way for precision combination therapy screening** https://bit.ly/3Yu5Ux0

## Introduction

Respiratory viruses cause major morbidity and mortality worldwide. RNA viruses, including the novel coronaviruses (severe acute respiratory syndrome coronavirus 2 (SARS-CoV-2)) and common seasonal viruses such as respiratory syncytial virus (RSV), result in serious respiratory infections. Seasonal RSV causes major morbidity, with an estimated 20 000 children aged <2 years admitted to hospital each winter in the UK [1, 2]. RSV is also a significant cause of morbidity and mortality in adults aged >60 years, causing annual epidemics in care homes during the winter season [3].

Experience from other viral infections has demonstrated that many successful therapies emerge from combining drugs with different mechanisms of action. For example, combination therapy has proved to be very successful for the treatment of hepatitis C virus [4–6], HIV [7], herpes simplex virus [8, 9], poliovirus [10], Ebola virus [11], Zika virus [12], and human cytomegalovirus [13]. Though there are compounds in development against RSV, some already in Phase II clinical trials, all of these are being evaluated as single therapies. The additive and/or synergistic effects of different drugs may be advantageous as they may improve efficacy, reduce the risk of resistance emerging, reduce the drug doses required and improve therapeutic risk-to-benefit ratio [14].

Small-animal models have been shown to have limited utility for clinically relevant investigations into RSV therapies due to the lack of complete RSV replication and symptomatic disease [15]. Screening in disease-relevant human tissue is a key component of modern drug discovery [16]. Many respiratory viruses, including RSV [17, 18] and SARS-CoV-2 [19, 20], have been shown to target human respiratory mucosecretory and ciliated cells for infection. Ciliated cells line the respiratory tract and possess motile cilia that help to clear pathogens and debris from vulnerable cells. However, these cells are not present in standard cell-line cultures, which lack the donor-specific variability seen in primary human tissue. This variability is crucial, as individual differences in cilia function and immune response can influence infection risks and drug efficacy. Therefore, using primary differentiated cell cultures derived from different human donors provides a more representative model for assessing drug efficacy.

**FIGURE 1:**
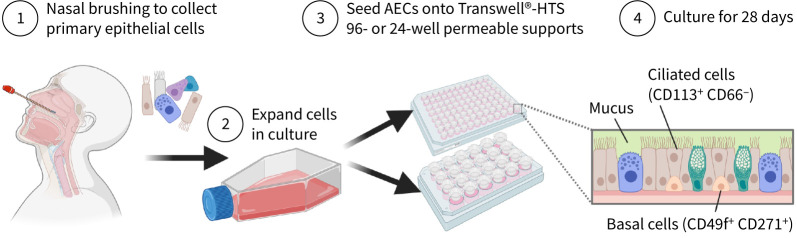
Graphical abstract of the model and method used. AEC: airway epithelial cell. Figure created with BioRender.com.

Air–liquid interface (ALI) cell culture is an established method for the growth of differentiated primary human airway epithelial cells for drug screening. We have developed a human primary cell culture method that allows extensive propagation of airway basal progenitor cells [21]. This method increases the numbers of differentiated cultures that can be grown from a single biopsy and makes 96-well high-throughput screening (HTS) ALI culture feasible [22]. While previous work demonstrated the potential of 96-well ALI platforms for large-scale antiviral drug screening [23], our study takes a complementary approach by focusing on ciliary functional read-outs and a novel analysis pipeline. Here we use a Transwell-96 *in vitro* assay of differentiated human ciliated epithelial cells as a method to test the efficacy of combination therapy using small molecule inhibitors of RSV (a schematic of the method is shown in figure 1) [22].

## Materials and methods

### Subjects

Informed written consent was obtained from all participants prior to enrolment in the study. Nasal epithelial brush biopsies were collected from healthy adult participants. Ethics approval for the study was provided by the UCL Living Airway Biobank (REC reference 14/NW/0128) or UCL Research Ethics (reference 4735/001).

### Culture and differentiation of human airway epithelial cells at the ALI using HTS Transwell-96 permeable supports

Nasal brush biopsies were received on ice in a transport medium which consisted of medium 199 (Life Technologies; #22340020) containing 100 U·mL^−1^ of penicillin, 100 µg·mL^−1^ of streptomycin (Gibco; #15290026), 25 µg·mL^−1^ of amphotericin B and 20.5 µg·mL^−1^ of sodium deoxycholate (Gibco; #15290018). Cells were plated for expansion directly into co-culture with mitomycin-inactivated 3T3-J2 fibroblasts as previously described [21, 22, 24]. Briefly, basal epithelial cells were cultured at 37 °C and 5% carbon dioxide until confluence and then separated from feeder cells using differential trypsinisation [21]. Basal cells were then seeded on collagen I-coated, semipermeable membrane supports in submerged culture in serum-free Airway Epithelial Cell Growth Medium (Promocell, Heidelberg, Germany). For 24-well Transwells (Corning 0.4 µm pores), 1×10^6^ cells were seeded per membrane in 250 µL of medium. For HTS Transwell-96 permeable supports (Corning, 3380, 1 µm and 0.4 µm pores, polyester membrane), 0.2×10^6^ cells were seeded per membrane in 75 µL of medium. After 24–48 h, apical fluid was removed, and cells were fed only from the basolateral side with serum-free Airway Epithelial Cell Growth Medium. The medium was exchanged three times per week for 28 days and the apical side was gently washed weekly with medium to remove mucus.

### Immunofluorescence staining and microscopy

The Transwell membrane was incubated in 4% (w/v) paraformaldehyde for 30 min at room temperature. Cells were stored at 4 °C in PBS until the time of staining. Cells were blocked and permeabilised using a blocking buffer (3% bovine serum albumin (BSA) in PBS containing 0.01% Triton X-100) at room temperature for 1 h, prior to overnight staining with a primary antibody (in 1% BSA in PBS) at 4 °C. The primary antibodies used were anti-β tubulin (Abcam, ab15568; 1:100) to detect ciliated cells and anti-MUC5AC (Invitrogen; 1:100) to detect mucin. Cells were washed three times in PBS for 5 min, and a secondary antibody (in 1% BSA in PBS; Molecular Probes; Alexa Fluor dyes) was applied for 2 h at room temperature. Hoechst 33258 staining solution (Sigma) was applied for 20 min at room temperature as a nuclear counterstain prior to imaging. For high magnification imaging, cells were mounted in an 80% glycerol and 3% n-propyl gallate (in PBS) mounting medium and images were obtained using an inverted LSM 710 confocal microscope (Zeiss).

### Flow cytometric evaluation of cell populations

At day 28 post ALI (time point chosen for maximal differentiation), cell cultures were washed with PBS. Accutase (Gibco, Thermo Fisher Scientific) was added to the apical and basal sides and cultures incubated at 37 °C for 15 min. DMEM containing 10% fetal bovine serum was added to the cell suspension, and then centrifuged at 400×*g* for 5 min. Cell pellets were resuspended in a fluorescence-activated cell-sorting (FACS) buffer (PBS, 1% BSA, 0.05 mM EDTA) containing Fc receptor blocker (BioLegend) for 5 min on ice. Cells were then centrifuged again at 400×*g* for 3 min and resuspended on ice for 20 min in a FACS buffer containing antibodies against the basal cell markers integrin α6 (CD49f-PE; clone GoH3; BioLegend) and anti-nerve growth factor receptor (NGFR/CD271-BV421; clone C40-1457; BD Biosciences) and cell markers CD66c (clone ASL-32; BioLegend) for secretory epithelial cells and promonin-1 (Prom1/CD133; clone AC133; Miltenyi Biotec) as a marker for ciliated cells [25]. After centrifugation at 400×*g* for 3 min, the cell pellets were resuspended in PBS containing viability dye (BioLegend) for 10 min on ice. Viability dye was removed by centrifugation at 400×*g* for 3 min and cells washed once in the FACS buffer before resuspending for analysis. Cells were run on a BD LSR II Flow Cytometer (BD Biosciences) and the results analysed using FlowJo 10 (FlowJo LLC).

### Transepithelial electrical resistance

Transepithelial electrical resistance (TEER) values were measured using an EVOM2 resistance meter and EndOhm chamber with a 6-mm culture cup for 24-well Transwells or a 1.5-mm electrode (STX100) designed for the HTS Transwells (World Precision Instruments). Readings were taken once per week for up to 4 weeks post ALI.

### Automated, high-content screening for drug efficacy and toxicity using HTS Transwell-96 permeable supports

**FIGURE 2:**
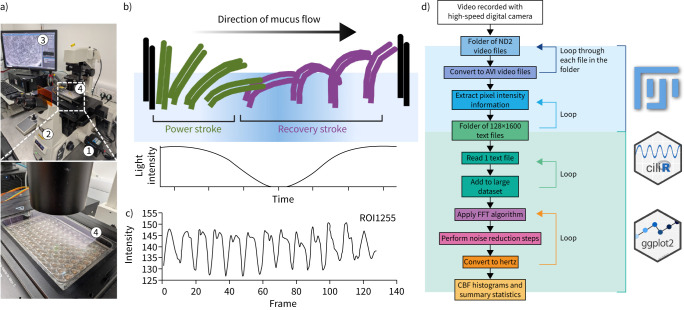
Quantifying ciliary beating from high-speed videos. a) Recording set-up. High-speed camera (1) mounted on an inverted microscope (2) records fields of differentiated airway epithelium cultured in a multi-well plate (3) seated in a stage adapter (4). b) Schematic of the ciliary beat cycle and the signal it produces. Coordinated power and recovery strokes propel mucus in the direction of flow (arrow), yielding a periodic intensity/contrast waveform over time (illustrative trace below). c) Representative raw time series extracted from a single region of interest (ROI). Oscillations correspond to ciliary strokes; the dominant frequency of this trace is taken as the ciliary beat frequency (CBF). d) Analysis pipeline. Videos acquired at high frame rate (≥100 fps) are imported into FIJI/ImageJ, ROIs are defined/cropped and per-ROI pixel intensity is measured across frames. Signals are optionally smoothed/detrended and transformed (fast Fourier transformation (FFT)) to identify the dominant peak (CBF). Results are collated into summary tables and visualised in R (*e.g.*, ciliR, ggplot2) to generate CBF histograms and per-sample summaries. AVI: Audio Video Interleave.

The recombinant green fluorescent protein (GFP)-tagged RSV A2 strain was kindly provided by Fix *et al.* [26] and propagated using HEp-2 cells for 3–5 days in Opti-MEM. The virus was purified as described previously [27], collected in BEBM (Life Technologies) and frozen at −80 °C. For ALI culture infection, the apical surface of the ALI cultures was rinsed with medium (BEBM), and 50 µL of viral inoculum (multiplicity of infection: 1) in BEBM was applied to the apical surface for 1 h at 37 °C and then removed. Following infection, cells were fed basolaterally with media containing different concentrations of drugs: an inhibitor of RSV Fusion (F) protein (CPD23, compound 23 from Cockerill *et al*. [28]) that blocks viral entry to the cell, and a polymerase inhibitor (ALS-8112, MedChemExpress) that causes lethal virus mutagenesis or disturbance of viral RNA synthesis to inhibit viral replication [29]. ALS-8112 was initially dissolved in dimethyl sulfoxide and made up to a working concentration range of 500–4000 nM in Airway Epithelial Cell Growth Medium; 153±76 nM was previously shown to result in a median inhibitory concentration (IC_50_) in RNA replication of RSV A2 in HEp-2 cells [29]. CPD23 was used at 5–80 nM (expected IC_50_ 1.59 nM (Elaine Thomas, Anti-infectives Unit, Pfizer, UK; personal communication)). Cells were monitored daily over a 7-day period using an inverted microscope system (Nikon Ti-E) with a 20× objective (figure 2a). Image acquisition was automated using the NIS-Elements JOBS (Nikon) module (see supplementary material) to measure GFP+ fluorescence (as an indicator of viral replication) and fast time-lapse (100 fps) recording (for ciliary beat frequency (CBF) calculation, as a sensitive indicator of cell toxicity) [30–32].

### Data analysis and statistical methods

CBF was determined from time-lapse files by first extracting the average pixel intensities (figure 2b,c) using ImageJ (National Institutes of Health) and, second, by performing a fast Fourier transformation (FFT) on these data using R (figure 2d); 6400 regions of interest (ROI), each with an area of 16.8 µm^2^, were analysed per file (pixel resolution=0.32 µm). The ImageJ macro and R code (ciliR package [33]) are available on GitHub (https://github.com/smithlab-code/ciliR). The ciliR package was previously [33] compared directly with previously published software ciliaFA [30]. Here ciliaFA and ciliR identified active cilia in 2247 out of 4803 and 2124 out of 4803 ROIs, respectively, with 126 (2.06%) excluded by ciliR quality control. Excluding these, both methods were nearly identical (r=1), with only a small fraction exceeding detection limit (shown in supplementary figure 1). Development of the code and analysis has been previously described [33].

Analysis of RSV-GFP fluorescence was performed using a custom ImageJ macro which counted the number of infected cells based on binary thresholding (macro included as supplementary material). Statistical analyses were performed in R version 4.0.2 [34] and GraphPad Prism version 9.0 using the statistical tests indicated in figure 5. Drug combination was analysed using the SynergyFinder version 3.14.0 package for R [35]. The Loewe model was applied to determine synergy score. The degree of smoothing in probability density estimates was determined *via* cross-validation.

## Results

### Evaluation and quality control of the ALI primary differentiated HTS Transwell-96 model

**FIGURE 3:**
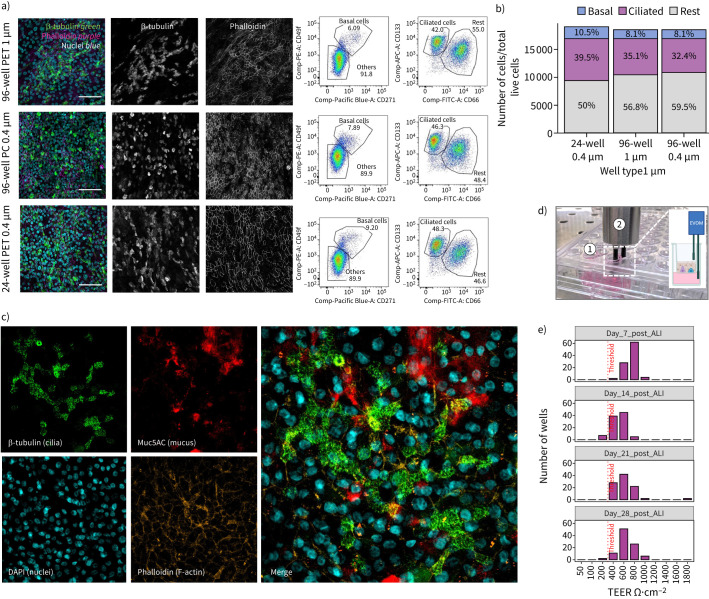
Characterisation of the 96-well high-throughput screening (HTS) air–liquid interface (ALI) culture model. a) Comparison of 24-well and 96-well HTS Transwell systems. Left panels show immunofluorescence images showing the presence of β-tubulin-expressing cilia (green), with phalloidin (purple) staining F-actin and DAPI (blue) staining nuclei. These images are representative of two healthy donors (three images). Right panels show flow cytometric evaluation of cell populations recovered from cultures grown at ALI on 96-well (data from four Transwells) and 24-well plates (data from two Transwells). b) Quantification of flow cytometric data showing different cell populations recovered from 96-well or 24-well plates. c) Representative immunofluorescence images showing the presence of β-tubulin-expressing cilia (green) and MUC5AC-expressing mucosecretory cells (red) in the 96-well HTS plate format ALI cultures. Phalloidin (orange) stains F-actin and DAPI (blue) stains nuclei. These images are representative of two healthy donors (three images). d) The set-up for manually measuring transepithelial electrical resistance (TEER) using the 96-well HTS system. (1) 96-well HTS plate and (2) EVOM probe. Insert created with BioRender.com. e) Histograms of the TEER of all wells in a 96-well HTS plate of ALI culture across the 4 weeks needed for differentiation, n=96.

We compared epithelial cell populations from two adult donors cultured in the 96-well HTS format with those cultured in the conventional 24-well Transwell system. Immunofluorescence imaging confirmed confluent epithelia with similar levels of ciliated cells (β-tubulin) and F-actin (phalloidin) (figure 3a). Flow cytometry showed no significant differences in basal (8.1% (96 wells) *versus* 10.5% (24 wells)) or ciliated cell proportions (32.4% (96 wells) *versus* 39.5% (24 wells)) between formats (figure 3a,b). Immunofluorescence imaging showed the expression of mucosecretory cells (MUC5AC+) within the confluent ciliated epithelium (figure 3c). To assess the uniformity of the 96-well HTS format, TEER was measured as the cells differentiated over 28 days (figure 3d,e). The decline in TEER after day 7 is consistent with maturation and differentiation dynamics in these cultures. Days 7–28 post ALI, 97–100% of wells had a TEER of >300 Ω·cm^−^^2^ (median=600 Ω·cm^−^^2^), which is regarded as sufficient for effective barrier function in quality control tests.

### Analysis of baseline ciliary beat frequency across the 96-well HTS plate

**FIGURE 4:**
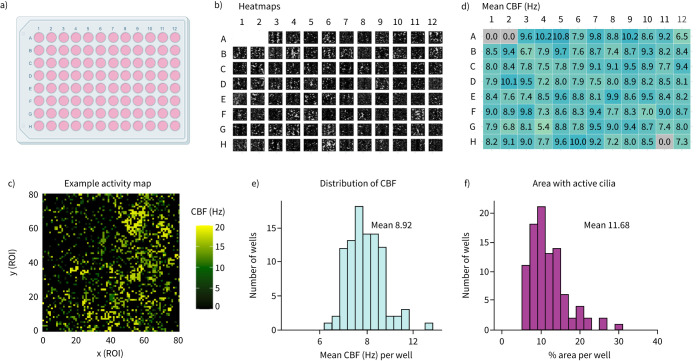
Quantification of ciliary function in 96-well high-throughput screening (HTS) air–liquid interface (ALI) cultures. a) Example layout of the 96-well HTS plate used for automated acquisition. b) Heatmaps summarising the fraction of the field containing motile cilia (active area) across the plate. c) Example activity map representing a single well showing region of interest (ROI)-level ciliary beat frequency (CBF) in hertz (colour bar). d) Plate map of mean CBF for each well (values in cells; colour scale). e) Histogram showing the distribution of mean CBF across the 96 wells. f) Distribution of the per cent area with active cilia per well. Data represent distribution of ciliary activity between plate wells, from the same donor.

We utilised automated well scanning to capture fast time-lapse videos of each well in the 96-well HTS plate, with cultures from the same donor (figure 4a). Using custom R analysis, we computed two read-outs per well: the per cent area with active cilia and the CBF. Active area is summarised across the plate as heatmaps (figure 4b), and as an ROI-resolved per-well activity (figure 4c). This method serves as a further quality control measure to identify and eliminate suboptimal wells (below TEER threshold and no active cilia at baseline), while also establishing the standard deviation in CBF across the plate. We also plotted the ciliary beating in the layout of 96-well plate (figure 4d), to establish baseline well variation (figure 4e,f) for use in analysis of subsequent drug and virus experiments.

### Assessment of drug efficacy and ciliary beat frequency under antiviral drug treatment

Next, we evaluated the 96-well HTS ALI model using a recombinant RSV with a GFP reporter linked to the L protein (polymerase) [26] to provide sensitive high-throughput read-outs of drug efficacy by identifying infected cells. We investigated the effect of antiviral drugs that inhibit different stages of the RSV replication cycle: an inhibitor of RSV Fusion (F) protein (CPD23) that blocks viral entry to the cell, and a nucleoside inhibitor (ALS-8112) that interferes with viral replication [29].

**FIGURE 5:**
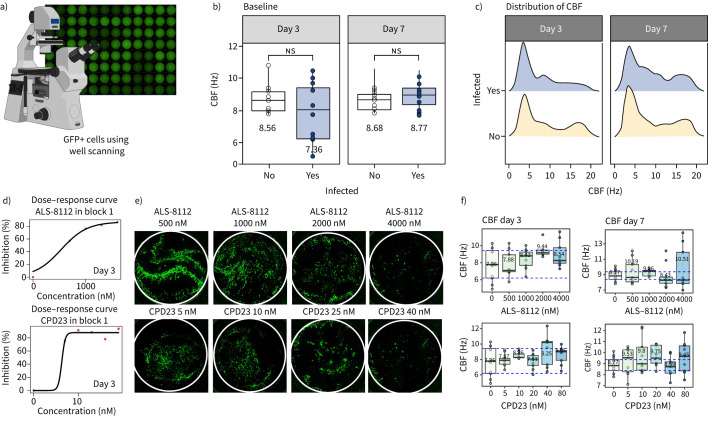
Viral inhibition and ciliary beat frequency (CBF) analysis with high-dose monotherapies of ALS-8112 and CPD23. a) Each well of 96-well high-throughput screening (HTS) air–liquid interface (ALI) culture was scanned for respiratory syncytial virus (RSV)-green fluorescent protein (GFP) fluorescence at days 3 and 7 post infection. b, c) Box plots and histograms showing the mean CBF and the distribution of CBF for ciliated cells with and those without RSV infection (paired t-test, ns: nonsignificant) per well (n=9). d) Dose–response curves showing inhibition of RSV-GFP by ALS-8112 and CPD23 (three plates). e) Representative fluorescence images of whole wells showing GFP expression, indicating RSV infection, at various concentrations of ALS-8112 (500–4000 nM) and CPD23 (5–80 nM). Wells treated with increasing concentrations of ALS-8112 and CPD23 exhibit reduced GFP expression, indicating inhibition of RSV-GFP. Scale bar represents 1 mm. f) Box plots showing the mean CBF and the distribution of CBF for ciliated cells treated with varying concentrations of ALS-8112 (500–4000 nM)) or CPD23 (5–80 nM) for 3 days (left panels) or 7 days (right panels) postinfection. No significant differences were detected (Wilcoxon test). ns: nonsignificant.

Three days after RSV infection, whole-well scans (figure 5a) were conducted and GFP fluorescence was measured to indicate the number of infected cells. We found that RSV infection alone did not affect CBF at day 3 or day 7 post infection (figure 5b,c). Treatment with the drugs as monotherapies showed an efficacy range of 57–98% GFP inhibition (n=1 donor, 3×3 replicates). The IC_50_ of ALS-8112 averaged 475 nM, aligning with literature values of 15–500 nM in similar ALI culture assays [36] and 20 nM in replicon assays [37]. The IC_50_ of CPD23, at 2.39 nM, was found to be lower than the lowest concentration tested (5 nM), and so is reported with lower confidence (figure 5d,e).

Focusing on cell toxicity, we found that high concentrations of ALS-8112 did not affect (p>0.05) the CBF, with a mean±sd CBF of 7.36±4.7 Hz at 0 nM and 8.54±5.09 Hz at 4000 nM at day 3 (figure 5f), and 8.77±5.08 Hz at 0 nM and 10.5±5.37 at 4000 nM (±2.1 Hz) at day 7 (figure 5f). Treatment with CPD23 also did not affect the CBF, with 8.64±5.11 Hz at the highest concentration of 80 nM on day 3 (figure 5f) and 9.45±5.43 Hz at day 7 (figure 5f).

### Evaluation of combination antiviral therapy in 96-well HTS: drug synergy and toxicity analysis

An advantage of the 96-well HTS ALI model over the 24-well system is the capacity to test multiple drugs or combinations, allowing evaluation of the combined antiviral effect of ALS-8112 and CPD23 over greater dose ranges. We generated a dose–response matrix from percentage inhibition compared with the untreated control (three replicates per plate completed in triplicate three plates) (supplementary figure 2A,B).

**FIGURE 6:**
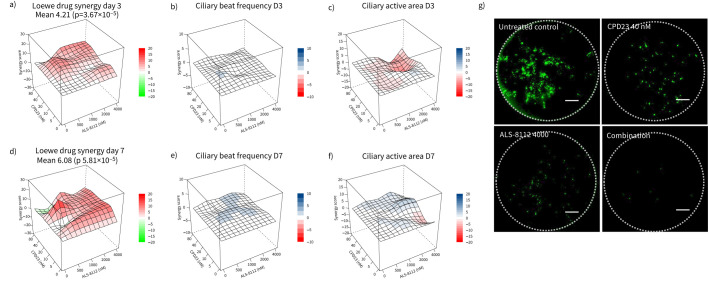
Dose–response and toxicity analysis of combination therapies in primary human airway epithelial cells. a) Synergy analysis at day 3 and d) day 7. The 3D synergy maps illustrate the interaction between ALS-8112 and CPD23. The synergy scores were calculated using the Loewe model, where red regions indicate synergistic interactions, and green regions indicate antagonistic interactions. Overall values between −10 and 10 are considered additive. b, e) Toxicity matrices of the ciliary beat frequency (CBF) across the 96-well high-throughput screening (HTS) treatment plate at b) day 3 and e) day 7, shown as a 3D surface plot. The mean CBF remained between 8 and 9 Hz, indicating minimal impact on ciliary activity. c, f) The active area percentage, indicating overall cellular activity, remained consistent at c) day 3 and f) day 7 with untreated plates across different concentrations of ALS-8112 and CPD23, demonstrating no significant toxicity. g) Representative fluorescence images of whole wells showing green fluorescent protein (GFP) expression, indicating respiratory syncytial virus (RSV) infection, with ALS-8112 (4000 nM) and CPD23 (40 nM) and in combination. Wells treated with ALS-8112 and CPD23 exhibit reduced GFP expression, indicating inhibition of RSV. Scale bar represents 1 mm.

Analysis of drug combination using the SynergyFinder package for R and Loewe model indicated an additive antiviral effect, with Loewe synergy scores of 4.21 (day 3) and 6.08 (day 7) (figure 6a,d). This software applies reference models [35, 38] for synergy score calculation that can be interpreted as the average excess response due to drug interactions beyond assumption. Values below −10 indicated that the effect is antagonistic, between −10 and 10 was considered additive and above 10 indicates that the drugs’ effect was likely to be synergistic [35]. The 3D synergy maps (figure 6a,d and supplementary figure 2C) highlight the synergistic and antagonistic dose regions with gradients of red and green, respectively. Representative GFP images are shown in figure 6g. Synergy maps for additional Bliss, Zero Interaction Potency (ZIP) and Highest Single Agent (HSA) models are shown in supplementary figure 2C.

We found that, used in combination, ALS-8112 and CPD23 showed an additive, but not synergistic, average antiviral effect when used in a range of 0–4000 nM (ALS-8112) and 0–80 nM (CPD23). The most effective combination was 500 nM ALS-8112+40 nM CPD23, both at day 3 and day 7 post RSV infection. The least effective combination (local minimum) was 500 nM ALS-8112+10 nM CPD23. These findings demonstrate the proof of principle for the system.

We also evaluated the effect of the combination therapy on ciliary activity, as an indication of cellular toxicity. We found that the CBF remained stable across treatments, indicating low toxicity. Mean CBF was 8.57 Hz (day 3) and 8.92 Hz (day 7) (supplementary figure 3). 3D plots comparing antiviral activity and CBF (figure 6b,e**)** support the model's suitability for functional screening.

## Discussion

This study demonstrates a cost-effective, high-throughput method for assessing drug efficacy and cellular toxicity using primary ciliated airway epithelial cells and standard inverted microscopes. By integrating novel analysis packages with a 96-well Transwell ALI culture system, we maximised experimental throughput while maintaining epithelial cell composition with similar numbers of total live cells and progenitor and differentiated cell populations as recovered from cultures grown using the conventional 24-well Transwell format. As we previously reported [22], there was no difference in the number of motile cilia and average CBF between the two culture formats. In this study we used cells from two donors, but future work will need additional donor validation (four or five independent donors).

Using an RSV-GFP reporter system, we evaluated two antiviral compounds targeting distinct stages of RSV replication: CPD23 [28] (fusion inhibitor) and ALS-8112 [29] (nucleoside analogue). Both drugs showed expected potency [36], with CPD23 approximately 100-fold more effective than ALS-8112. Importantly, neither drug impaired CBF, even at concentrations far exceeding their IC_50_ (13- or 60-fold), indicating low cellular toxicity [39]. These measurements were obtained using an automated widefield fluorescence microscope coupled with high-speed video microscopy, enabling medium-throughput functional screening of multiple plates within a 2–3-h window.

We showed that ALS-8112 and CPD23 result in an average additive antiviral effect for combinations using a range of 0–4000 nM (ALS-8112) and 0–80 nM (CPD23), with a peak of maximum additive effect for 500 nM of ALS-8112 combined with 40 nM of CPD23 [35]. We used the Loewe model to assess the synergistic (score >10), additive or antagonistic (score <−10) effect of drug combinations. A limitation of this method is that it cannot directly assess a combination effect that is higher than the achievable effect of the individual drugs, as the concentrations of the inhibitors used alone already resulted in a >50% reduction in RSV-GFP. Ideally, combination analyses should be performed at concentration ranges spanning each compound's IC_50_, which is more achievable with the greater number of experimental units available with the 96-well HTS system. Future studies will be directed to address this limitation.

We also recognise that reliance on CBF, while valuable as a noninvasive, non-end-point functional read-out, is limited when used as the sole indicator of toxicity [40]. Complementary assays assessing apoptosis and viability (*e.g.*, lactate dehydrogenase release, caspase activity) as well as molecular and metabolic data would provide a more comprehensive evaluation of cytotoxicity for future studies. Furthermore, ultrastructural analysis by transmission electron microscopy, as we have previously reported [17], could provide additional validation – both in confirming epithelial differentiation quality and in detecting early ciliary alterations associated with RSV infection or drug treatment. Together, these follow-up approaches will be essential to extend mechanistic understanding beyond the methodological proof of principle established in this study.

### Conclusion

We have shown that 96-well HTS ALI cultures can be used to model the airway epithelial response to RSV infection during antiviral therapy and have demonstrated application of a novel analysis pipeline. We anticipate that this system will be widely applicable to other respiratory viruses, such as SARS-CoV-2 and influenza A virus. In the future, larger-scale combinatorial screening of drug therapies using models derived from vulnerable groups such as infants and patients with primary immunodeficiencies has the potential to rapidly inform the clinical translation of effective drug regimens targeting respiratory RNA viruses.

## Data Availability

Custom code for the analysis performed in this study is publicly available *via* GitHub at https://github.com/smithlab-code/ciliR.

## Supplementary material

**Please note:** supplementary material is not edited by the Editorial Office, and is uploaded as it has been supplied by the author. Supplementary material 01283-2025.SUPPLEMENT Figure S1 01283-2025.SUPPLEMENT4 Figure S2 01283-2025.SUPPLEMENT2 Figure S3 01283-2025.SUPPLEMENT3
