# Supplementary figures and images for: Donor-derived airway air–liquid interface model for high-throughput screening of antiviral combinations with concurrent analysis of antiviral efficacy and epithelial toxicity using ciliR
Source: ERJ Open Res. 2026 Jun 1;12(3):01283-2025. doi: 10.1183/23120541.01283-2025 (PMC13224156; doi:10.1183/23120541.01283-2025)

Supplementary Figure 1

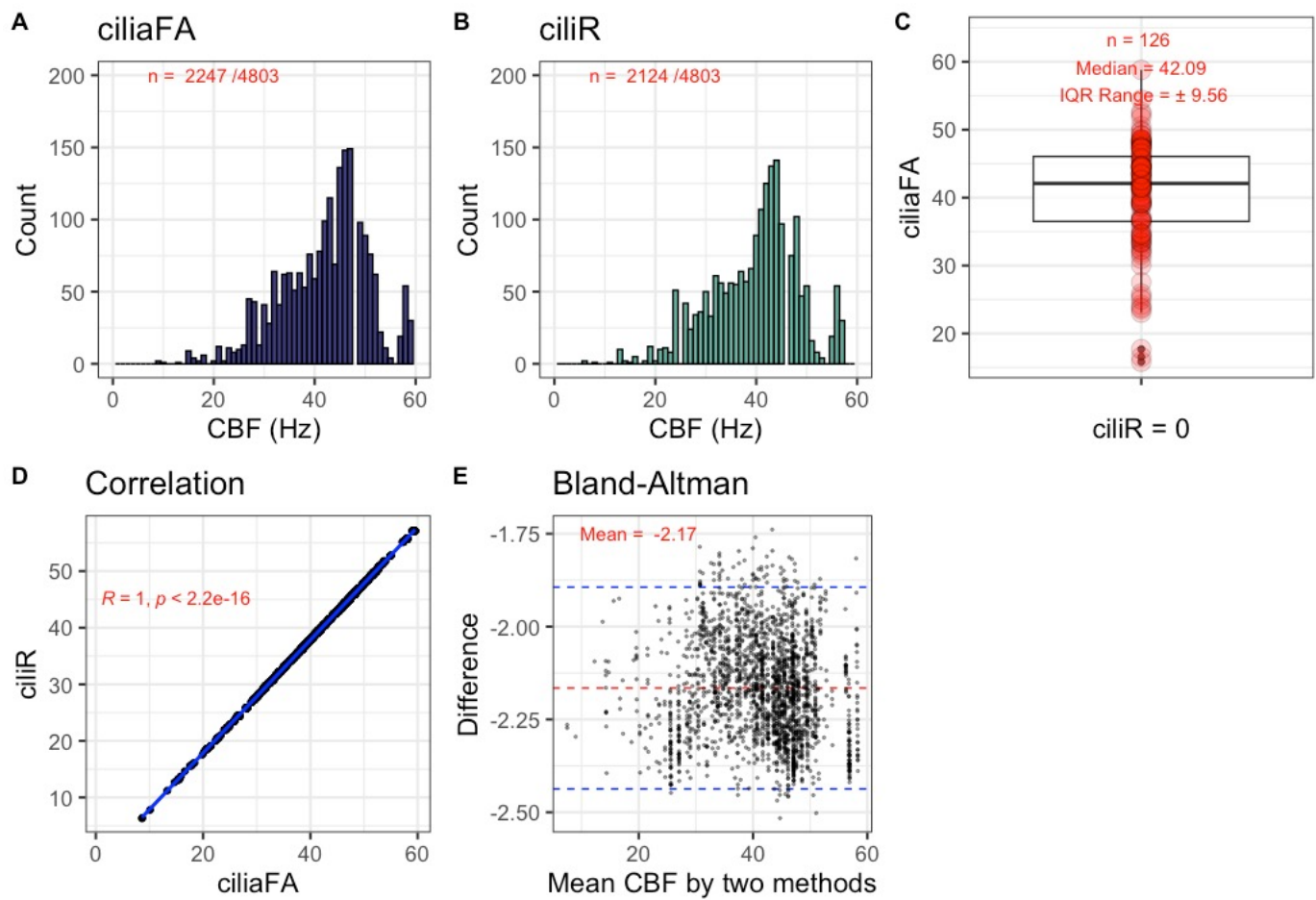

Supplement: Supplementary file 2 [file 01283-2025.SUPPLEMENT4.pdf]

Supplementary Figure 2

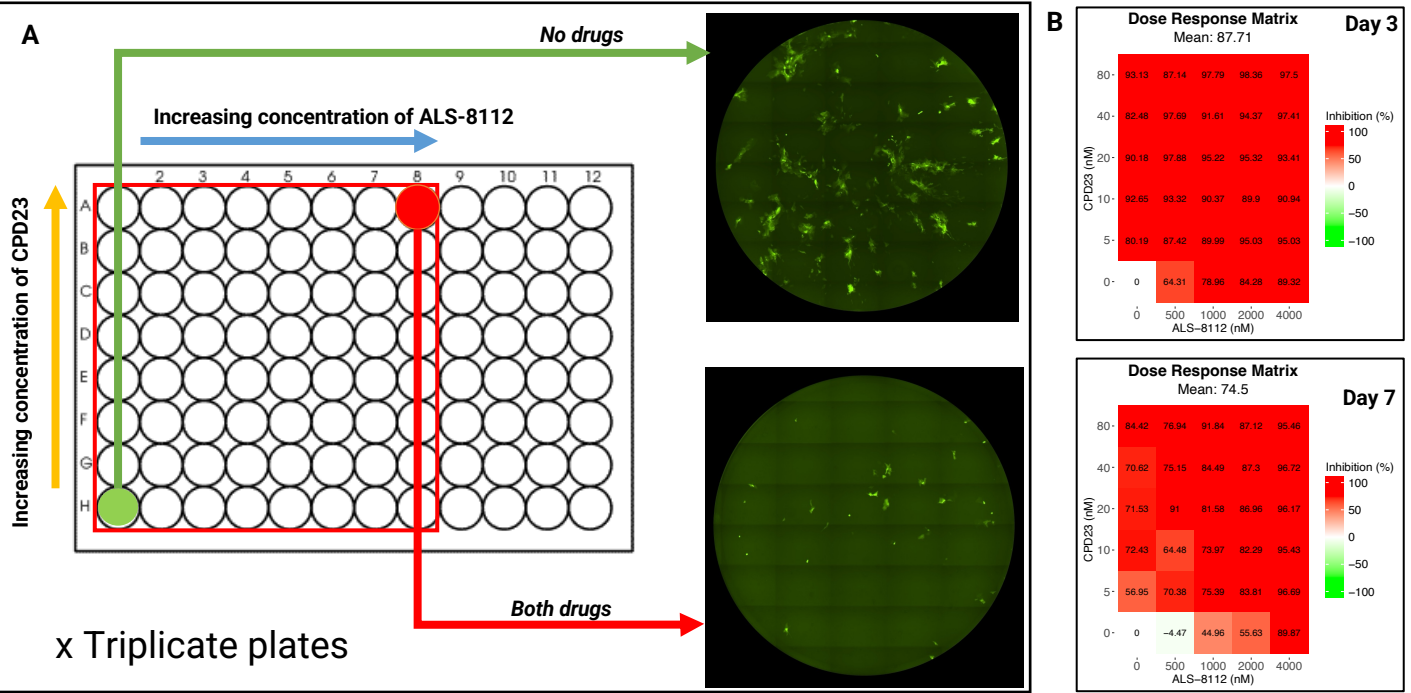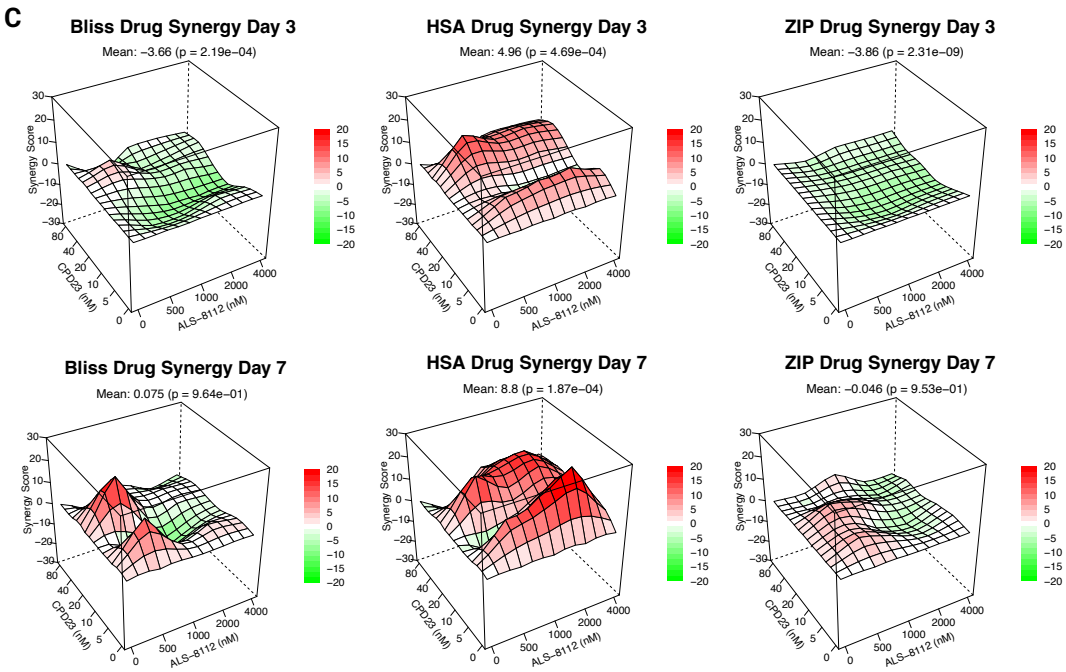

Supplement: Supplementary file 3 [file 01283-2025.SUPPLEMENT2.pdf]

Supplementary Figure 3

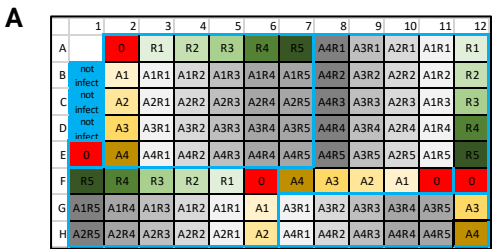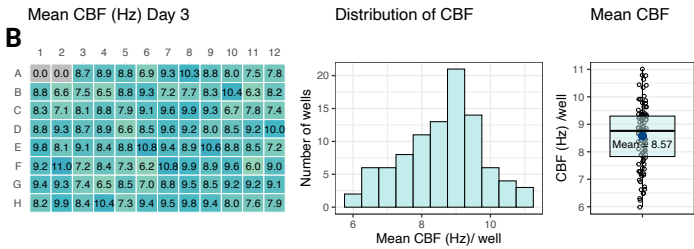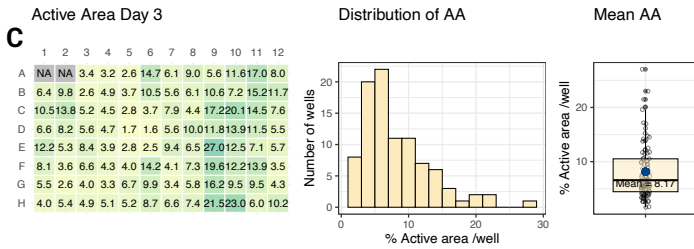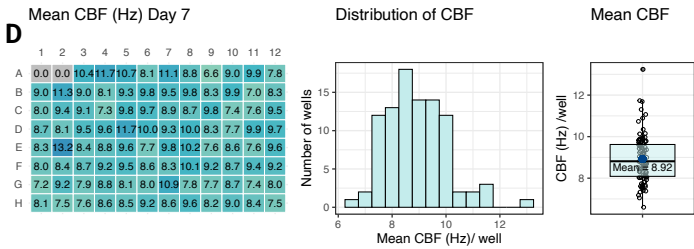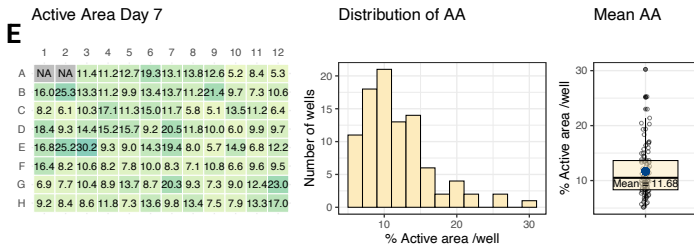

Supplement: Supplementary file 4 [file 01283-2025.SUPPLEMENT3.pdf]
